# A comparison of two psychiatric service approaches: findings from the Consultation vs. Liaison Psychiatry-Study

**DOI:** 10.1186/s12888-016-1171-4

**Published:** 2017-01-10

**Authors:** Caroline Lücke, Jürgen M. Gschossmann, Alena Schmidt, Juliane Gschossmann, Alexandra Philomena Lam, Charlotte Elizabeth Schneider, Alexandra Philipsen, Helge H. Müller

**Affiliations:** 1 Medical Campus University of Oldenburg, School of Medicine and Health Sciences, Psychiatry and Psychotherapy – University Hospital, Karl-Jaspers-Klinik, Hermann-Ehlers-Strasse 7, Bad Zwischenahn, D-26160 Germany; 2 Department of Internal Medicine, Klinikum Forchheim, Forchheim, D-91301 Germany; 3 Heinrich Heine University Düsseldorf, Düsseldorf, D-40225 Germany; 4 Department of Psychiatry and Psychotherapy, Friedrich-Alexander-University Erlangen-Nuremberg, Erlangen, D-91054 Germany

**Keywords:** Consultation psychiatry, Liaison psychiatry, Psychiatric treatment

## Abstract

**Background:**

Psychiatric comorbidities are common in somatically ill patients. There is a lack of data that can provide clear insights into substantial comparative advantages of different Consultation/Liaison Psychiatry (CLP) services.

**Methods:**

The Consultation versus Liaison Psychiatry-Study collected and analyzed data of 890 primarily somatically ill hospital inpatients presenting with psychiatric symptoms in a prospective observational study design. One group was treated via a liaison-model (LM) with regular consultation hours, the other via an on-demand-model (ODM) with individually requested consultations.

**Results:**

Five hundred forty-five LM and 345 ODM patients were compared. Patients in the LM were, on average, older compared to the patients of the ODM. The vast majority (90.8%) of individuals for whom a psychiatric consultation was requested came from internal medicine. The most common diagnoses were affective disorders (39.3%), organic mental disorders (18.9%), alcohol-induced mental disorders (11.3%) and reactions to severe stress/adjustment disorders (10.4%). Organic mental disorders were significantly more common in patients seen in the LM (24.0% vs. 10.3%, *p* < 0.001) while affective disorders were more frequently diagnosed in the ODM (46.6% vs. 34.8%, *p* = 0.001).

Patients seen in the ODM were, on average, more severely affected compared to patients seen in the LM and required more extensive treatment. 16.3% of ODM patients were regarded as potentially suicidal; among these, 3.5% were acutely suicidal and 12.8% latently suicidal. Any form of further treatment was required by 93.0% of ODM patients compared to 77.8% in the LM. Pharmacological treatment with benzodiazepines, usually used as short-term treatment, was more frequently prescribed to patients seen in the ODM while patients seen in the LM were more often started on selective serotonin reuptake inhibitors, indicative of long-term treatment.

**Conclusions:**

Patients in need of less acute treatment were considerably less common in the ODM. The data indicate a possible risk of such patients to remain unrecognized.

A *quasi-liaison* model is recommended to be the best suitable and cost-effective way of providing psychiatric care to somatically ill patients with psychiatric comorbidities.

## Background

Consultation-liaison psychiatry (CLP) is the discipline of providing professional psychiatric care to hospital patients of other medical disciplines who are primarily admitted for somatic reasons and in whom comorbid psychiatric symptoms become evident at admission or during the course of their hospital stay. It has long been known that psychiatric comorbidities in hospital patients are common and often serious [1, 2]. For example, Silverstone [3] found that 27% of medical inpatients could be diagnosed with a psychiatric disorder according to DSM IV criteria, and in the Lübeck General Hospital study, up to 46% of randomly selected hospital patients received a clinically based psychiatric diagnosis [4]. Over the years, providing efficient and timely psychiatric care to these patients has been shown to be beneficial with regard to various medical and socioeconomic parameters, including length of hospital stay, concordance and follow-up outcome [5, 6]. Psychiatric symptoms may be a direct expression of an organic disorder, an indirect effect of the burden of severe illness (e.g., in the form of affective or anxiety disorders), or totally independent of the somatic reason for the hospital stay. In somatoform disorders the psychiatric pathology is often the primary reason for the hospital admission [7]. The psychiatric diagnoses most frequently found in somatically ill hospital patients are affective disorders, organic mental disorders, somatoform disorders and alcohol abuse [8, 9]. Yet, different studies from different patient populations have produced different results, and results may also change over time. For example, a German study from 1995 found an alcohol-related diagnosis in 14.5% of medical patients [10], and alcoholism was among the most prominent diagnoses in the Lübeck General Hospital study [4]. However, alcoholism played only a minor role in recent studies from Italy and Australia [11, 12]. Thus, one aim of the present study was to provide updated data from a large Western European patient population on the distribution of psychiatric diagnoses in hospital patients. These data are not only of interest to clinical psychiatrists; they also have implications for the organizational improvement of healthcare conduct and are relevant to current socioeconomic questions of cost-effectiveness in consultation-liaison psychiatry, since it is known that effective measures of early recognition and treatment of psychiatric illness can not only improve patients well-being and social functioning but also reduce overall treatment costs [5, 13, 14].

The exact conduct and methodology of CLP have varied and developed over the past decades and across different countries with diverse healthcare systems. Historically, consultation-psychiatry has referred to the request for a second expert opinion by the somatically treating physician regarding a specific patient, while liaison-psychiatry referred to the integration of psychiatry within a somatic department. Liaison-psychiatry generally included regular psychiatric consultation hours as well as routine guidance and supervision of the somatically treating staff by the psychiatrist regarding psychiatric/psychosomatic themes [8, 15, 16]. While the latter model is certainly desirable, such an extensive approach is only rarely applied in European healthcare systems today, mainly for the economic concerns on the high costs of employing an own psychiatric team with sufficient work hours to ensure the above described tasks [16, 17], despite growing evidence that CL-services can be cost-effective, in example by reducing the length of hospital stay [5, 18, 19]. Still, for smaller-sized clinics, the appointment of an in-house liaison psychiatrist may indeed be unaffordable.

Yet, a “quasi –liaison” approach is often used in clinics that do not have an associated psychiatric department and therefore must request external psychiatric support. In such settings, it seems feasible and economically sensible to have regular in-house consultation hours conducted by an externally based psychiatrist. However, little is known about the effectiveness of this approach in identifying relevant psychiatric disorders in hospital patients and initiating adequate treatment. While this model could potentially disadvantage acutely affected patients, who might have to wait until the next scheduled appointment, it may actually benefit patients with less prominent psychiatric symptoms because the threshold for physicians to arrange a psychiatric assessment may be lower when there are regular consultation hours versus an active request for an “emergency” consultation. Thus, in such a setting, patients are more likely to receive routine psychiatric care even in the absence of obvious acute pathology, while such patients might be neglected in a purely on-demand approach [15]. Because medical inpatients all share the experience of a significant recent life-event – namely hospital admission [20] – a high prevalence of psychological distress is likely, even if it is not immediately expressed by the patients. This prevalence justifies the easy availability of psychiatric counseling.

Although the different styles of consultation-liaison have been quite extensively discussed, and there has even been rivalry between styles [15, 21–23], there is a lack of primary data that can provide clear insights into the substantial comparative advantages of different CLP models [24]. In this study, we systematically compared patient data from a traditional “consultation-model” in a large University Clinic with data from a “liaison-model” in a general hospital.

## Methods

### Study design and setting

In the Consultation versus Liaison Psychiatry-Study, we collected and analyzed data of primarily somatically ill hospital patients from two German hospitals who were assessed by psychiatrists for comorbid psychiatric symptoms. The study design was prospective and observational with no deviation from the routine care in the participating clinics. The necessity of a psychiatric evaluation was decided by the somatically treating physicians. Psychiatric consultations were performed according to two different systems, described below:

In the Klinikum Forchheim, a medium-sized general hospital, patients were seen in a “quasi-liaison-model” (LM), meaning that an externally based psychiatrist came into the clinic to see patients with psychiatric symptoms at two fixed times per week (Thursdays and Fridays). Usually, the maximum waiting time for a psychiatric assessment after a patient had developed symptoms was 4 days. In the rare cases that required immediate emergency psychiatric treatment, patients were transferred to a psychiatric clinic after a telephone consultation with the liaison psychiatrist. Deviating from the original idea of the liason-model, the psychiatrist was not part of the hospital’s medical team and did not regularly provide supervision of medical staff.

In contrast, for patients of the University Clinic Erlangen, psychiatric care was provided in an “on-demand-model” (ODM): an in-house psychiatric consultation for an individual patient could be requested at any time, and assessments were usually performed shortly after the request or even immediately in urgent cases. The psychiatrist was based at the psychiatry department associated with the University Clinic, which was located nearby, thus facilitating quick and economical consultations.

### Conduct of psychiatric consultations

All psychiatric consultations were performed by experienced psychiatrists, and more than 90% of consultations were performed by the same three psychiatrists. Consultations consisted of a detailed psychiatric and medical history and the assessment of current psychopathology as evidenced during the appointment. In addition, a third party anamnesis was usually obtained from the treating physician who had initiated the psychiatric consultation. A neurological examination was performed when applicable. There was no difference in the conduct of psychiatric consultations between the two clinics.

### Data sampling and study period

Demographic and clinical data, including age, gender, psychopathological symptoms, suicidal tendencies, medication, psychiatric diagnosis and treatment recommendations, were systematically collected for all patients who were psychiatrically evaluated within the timespan of data collection. Data were collected between March 2014 and September 2015 in the clinic running the LM and between Sept 2011 and April 2012 in the clinic running the on-demand-model. Patient numbers and demographic data are described in the results section.

Furthermore, the total number of patient admissions in the respective clinics during the time span of data collection was obtained from the hospitals’ controlling system to calculate the percentage of patients needing a psychiatric consultation among the population of hospital patients. Since the two clinics were differently structured, with the University Clinic Erlangen featuring a wider range of medical subspecialties which may have biased the result, we calculated this ratio for patients from the departments of internal medicine only.

### Data processing and analysis

All data were processed using SPSS statistics 23 and analyzed descriptively, or analyzed using the appropriate statistical method for comparisons between clinics (Chi-Square test for dichotomous data, *T*-Test for continuous data). To account for multiple comparisons, levels of significance were adjusted using a Bonferroni correction.

### Ethics and consent to participate

The study was approved by the local ethics committee of the Friedrich-Alexander-University of Erlangen-Nuremberg. All patients gave informed consent to participate after receiving an explanation of the study design and procedures by the psychiatrist performing the psychiatric consultation.

## Results

Data were collected for a total of 890 patients; of these, 545 were patients of the Klinikum Forchheim, where consultations were conducted in the LM, and 345 were patients of the University Clinic Erlangen who were seen in the ODM. The mean age was 64.66 years (range 15–99). Patients in the LM were, on average, older compared to the patients of the ODM (mean age 69 y. versus 57 y.). The ratio of male to female patients was nearly equal in both clinics, with female patients comprising approximately 60% of the total population. Regarding the somatic medical specialties where the patients were admitted, the vast majority (90.8%) of individuals for whom a psychiatric consultation was requested came from internal medicine (87.3% in the LM, 96.2% in the ODM). In the LM, an additional 8.1% of patients came from general or trauma surgery. Patients from other medical specialties were very rare, and thus a statistical analysis of the influence of specialties on other outcome parameters was not feasible due to the small sample sizes.

Total numbers of inpatients in the departments of internal medicine in the respective clinics during the time of data collection were 6990 in the LM and 8992 in the ODM, resulting in a proportion of patients requiring psychiatric consultation of 6.8% in the LM and 3.7% in the ODM (*p* < 0.001).

The psychiatric diagnoses given to patients, based on the psychopathology present at the time of examination, are shown in Table 1.

**Table 1:** Psychiatric diagnoses

		Hospital		Total
		Klinikum Forchheim	University Clinic Erlangen	
Organic mental disorder	Count	128	33	161
	% within hospital	24.0	10.3	
	% of Total			18.9
Alcohol induced mental disorder	Count	58	38	96
	% within hospital	10.9	11.9	
	% of Total			11.3
Mental disorder induced by drugs other than alcohol	Count	8	13	21
	% within hospital	1.5	4.1	
	% of Total			2.5
Psychotic disorder	Count	23	18	41
	% within hospital	4.3	5.6	
	% of Total			4.8
Affective disorder	Count	186	149	335
	% within hospital	34.8	46.7	
	% of Total			39.3
Phobic/other anxiety disorder	Count	42	24	66
	% within hospital	7.9	7.5	
	% of Total			7.7
Reaction to severe stress/adjustment disorder	Count	65	24	89
	% within hospital	12.2	7.5	
	% of Total			10.4
Dissociative and conversion disorders	Count	4	4	8
	% within hospital	0.7	1.3	
	% of Total			0.9
Somatoform disorder	Count	36	8	44
	% within hospital	6.7	2.5	
	% of Total			5.2
Eating disorder	Count	2	4	6
	% within hospital	0.4	1.3	
	% of Total			0.7
Sleep disorder	Count	3	0	3
	% within hospital	0.6	0.0	
	% of Total			0.4
Other psychiatric diagnosis	Count	10	11	21
	% within hospital	1.9	3.4	
	% of Total			2.5
No psychiatric disorder	Count	45	2	47
	% within hospital	8.4	0.6	
	% of Total			5.5
Total	Count	610	328	938

Multiple diagnoses were possible. Percentages are based on the number of patients with documented data on the psychiatric diagnosis (*n* = 853). Patients with missing data (*n* = 37) were excluded from the analysis

In the overall patient population, the most common diagnoses were affective disorders (39.3%), organic mental disorders (18.9%), alcohol-induced mental disorders (11.3%) and stress-related disorders (10.4%) as classified under the code F43 in the International Classification of Disease 10 (ICD-10). This category includes acute stress reactions, adjustment disorders and post-traumatic stress disorders. Organic mental disorders were significantly more common in patients seen in the LM (24.0% vs. 10.3%, *p* < 0.001) while affective disorders were more frequently diagnosed in the ODM (46.6% vs. 34.8%, *p* = 0.001). Reactions to severe stress/adjustment disorders and somatoform disorders were both more common in patients seen in the LM, but this result was not statistically significant after Bonferroni correction for multiple comparisons.

In 8.4% of patients seen in the LM, no psychiatric diagnosis was given as the result of the consultation, which was extremely rare in patients seen in the ODM (0.6%, *p* < 0.001).

In general, patients seen in the ODM were, on average, more severely affected compared to patients seen in the LM. A total of 16.3% of ODM patients were regarded as potentially suicidal; among these, 3.5% were acutely suicidal, meaning they could not distance themselves from acute suicidal intentions and 12.8% were latently suicidal, meaning suicidal thoughts in the absence of acute suicidal intentions. In the LM, only 1.3% of patients were acutely suicidal and 6.5% were latently suicidal. The differences between the clinics were highly significant (*p* < 0.001) (Table 2); 7.6% of patients seen in the ODM had a previous suicide attempt. In the LM, only 1.7% of patients had a previous suicide attempt.

**Table 2:** Suicidality

			Hospital		Total
			Forchheim	Uniklinik Erlangen	
Suicidality	None	Count	494	288	782
		% within Hospital	92.2	83.7	
		% of Total			88.9
	Latent	Count	35	44	79
		% within Hospital	6.5	12.8	
		% of Total			9.0
	Acute	Count	7	12	19
		% within Hospital	1.3	3.5	
		% of Total			2.2
Total		Count	536	344	880
		% within Hospital	100.0	100.0	
		% of Total			100.0

In the general hospital running the LM, during the time span of data collection a total of 10 patients were admitted after a failed suicide attempt. After treatment of somatic pathologies and telephone consultation with the liaison psychiatrist, these patients were immediately transferred to a psychiatric clinic. Because these brief telephone consultations did not allow for a detailed assessment, these 10 patients are not included in our data.

Patients seen in the ODM required more extensive further treatment following their consultations (Table 3): 93.0% required any form of further treatment compared to 77.8% in the LM. For 53% of patients in the ODM, inpatient psychiatric treatment was recommended, compared to only 20.7% of patients in the LM. Furthermore, for 28.7% of patients in the ODM, admission to the psychiatric inpatient crisis unit was considered necessary, while admission to a crisis unit was an extremely rare event for patients in the LM (0.4%). The differences between clinics were statistically highly significant (*p* < 0.001).

**Table 3:** Recommended further psychiatric treatment

		Hospital		Total
		Klinikum Forchheim	University Clinic Erlangen	
No further treatment	Count	118	16	134
	% within Hospital	22.2	7.0	
	% of Total			17.6
Outpatient psychotherapy	Count	27	6	33
	% within Hospital	5.1	2.6	
	% of Total			4.3
Outpatient psychiatric treatment	Count	250	62	312
	% within Hospital	47.1	27.0	
	% of Total			41.0
Outpatient psychiatric treatment and psychotherapy	Count	26	18	44
	% within Hospital	4.9	7.8	
	% of Total			5.8
Inpatient, open ward	Count	108	56	164
	% within Hospital	20.3	24.3	
	% of Total			21.6
Inpatient, crisis unit	Count	2	66	68
	% within Hospital	0.4	28.7	
	% of Total			8.9
Immediate emergency transfer to psychiatric unit	Count	0	6	6
	% within Hospital	0.0	2.6	
	% of Total			0.8
Total	Count	531	230	761
	% within Hospital	100.0	100.0	
	% of Total			100.0

Antidepressants were the most frequently prescribed medication following psychiatric consultations. Selective serotonin reuptake inhibitors (SSRI) were prescribed in 17.4% of patients, but there was a significant difference between the consultation models, with 23.5% of patients in the LM receiving SSRI versus only 7.8% in the ODM (*p* < 0.001). Prescription rates of SSRI before the psychiatric consultations were equally low in both clinics (7.9 and 7.8%).

In contrast, benzodiazepines were more often prescribed to patients in the ODM (38.6%) compared to patients in the LM (20.0%), while the pre-consultation prescription rates were again similar (9.0 and 9.8%).

The vast majority of the psychiatric consultations requested by the ward physicians of the respective clinical specialties proved to be justified. In 92.8% of the total patient population, the need for a psychiatric consultation was confirmed by the psychiatrist. In the ODM the rate of justified psychiatric consultations even reached 98.5%, versus 88.3% in the LM (*p* < 0.001).

## Discussion

The Consultation versus Liaison Psychiatry-Study involved a large sample size, providing up-to-date information from a Western European patient population. Methodologically, a particular strength of our data is the low inter-rater bias because nearly all patients were assessed by the same three psychiatric specialists. The prevalence rates of psychiatric diagnoses found in this study generally correspond well with data from the literature [4, 25], indicating that our data are a valid sample of the population in question. As shown in previous studies, our results confirm that affective disorders as well as adjustment disorders, organic disorders and alcoholism continue to be common comorbidities in somatically ill hospital patients.

The main goal of the study was to compare two systems of CLP, the “liaison-model”, with fixed patient appointments, and the “on-demand-model”, with individually requested consultations. While some of the results regarding diagnoses and recommended treatments were similar between the two models, there were also significant differences between the two populations of patients. Our finding, that the proportion of internal medicine patients, for whom a psychiatric consultation was requested, was significantly lower in the ODM than in the LM strongly suggests that the model of CL-psychiatry used in clinics influenced patient care. Several explanations for this fact can be concluded from our data:

In general, patients seen in the ODM seemed to be more acutely and severely affected, i.e., suicidality was more common in the ODM. If we account for the 10 acutely suicidal patients in the LM who were directly transferred to a psychiatric clinic and were thus not included in the data set, the rate of acute suicidality was equal in both models. Still, there was a significantly higher number of latently suicidal patients in the ODM. Additionally, the fact that further psychiatric inpatient treatment was recommended much more often for patients seen in the ODM, combined with the more frequent prescription of benzodiazepines in the ODM, hints at the “emergency” nature of many consultations in this model. In many cases, the emergent nature of a consultation may have meant that patients exhibited severe non-compliant or disruptive behavior, thus prompting the somatically treating physician to seek psychiatric help to ensure further somatic treatment and lessen immediate distress for both patient and staff [26]. In contrast, patients seen in the liaison model seemed to be, on average, more mildly affected. For the majority of these patients, further psychiatric treatment was recommended in an outpatient setting. It can be assumed that the model of two fixed psychiatric consultation hours per week encouraged the referring physicians to include those patients in their consultation requests, in whom a latent psychiatric pathology was suspected but was not prominent or interfering enough to justify an on-demand consultation. For example, in the LM a considerable proportion of patients seen during consultation hours suffered from dementia (10.6% of the patients), which often does not require acute treatment, while such patients were rare in the ODM (1.8%). Prescription of selective serotonin reuptake inhibitors following the consultation was significantly more common in patients seen in the LM, indicating that consultations in the LM focused more on patients’ long-term treatment and outcomes, which could be due to the availability of more time per patient in this “planned” setting compared to the “emergency” setting of the ODM. Thus, patients with milder psychiatric symptoms might actually have profited from the liaison setting, while for patients with more acute symptoms, the waiting time of up to 4 days until the next consultation hour might have been difficult for both patients and ward staff. Because, for security reasons, this waiting time was obviously not acceptable for high risk patients (such as those who had made an acute suicide attempt), the liaison psychiatrist was available for immediate telephone consultation in such emergencies. A quick transfer to a psychiatric clinic was then organized for these patients, who thus received appropriate and timely care.

Setting a lower threshold for patients to be seen by a psychiatrist, which likely occurred in the LM, could have the economic downside of creating more “unjustified” consultations. Indeed, while the indication for a psychiatric consultation was confirmed for nearly all patients seen in the ODM, 11.7% of patients seen in the LM did not receive a psychiatric diagnosis following the consultation. However, this rate still seems low when weighted against the risk of overlooking psychiatric pathology and the need for treatment, which could occur if the threshold for demanding a psychiatric consultation was set at a higher level.

Reviewing the advantages of both models as indicated by our data, an improved yet economically feasible approach might entail a combination of the two models, with both the possibility of quick on-demand consultations (in cases of acute pathology) and fixed consultation hours to address patients with less pronounced pathology and enable a more in-depth review beyond just the short-term treatment of symptoms.

It must be noted, however, that none of the models presented here could provide the full range of psychiatric care as sought in the traditional understanding of the liaison model. This model includes substantial training and counseling of ward staff, guidance on improvement of staff/patient interactions and routine screening of patients with symptoms of distress, thus promoting a more holistic view of the patient. Still, even such an extensive approach faces a variety of problems [27], most notably the resistance of medical physicians and staff, who may feel more patronized than helped by the constant surveillance of the liaison psychiatrist [12]. Such conflicts are less likely in the more small-scale models presented here because the CL psychiatrist’s presence is much more limited, and his role remains that of a consultant rather than a team member.

In general, discussing these aspects, it needs to be kept in mind that the theory of healthcare-delivery-models is a complex area, involving many layers, including clinical, economic, ethical and political questions, and quantitative measures, as provided here, may not be perfectly suited to address some of these aspects.

A limitation of the data presented here is the lack of direct measurements of efficacy [28], i.e., lengths of hospital stays, patient and staff feedback, concordance with treatment recommendations and follow-up data on the outcomes of patients. These data would be necessary to draw firmer conclusions about the benefits of these models of CLP for different groups of patients. Future studies on the role of the organizational setting in CLP should ideally include such direct outcome measurements.

A further limitation is the possible bias of our data due to differences in the patient populations of a medium-sized general hospital (LM) compared to a large University hospital (ODM). A bias introduced through different medical subspecialties seems unlikely because the vast majority of patients in both clinics came from internal medicine. However, University clinics might treat patients with rarer or more complicated illnesses. Additionally, the younger average age of patients seen in the ODM could potentially influence the results, particularly regarding the proportion of dementia. Gender-bias can be excluded because the ratio of male to female patients was nearly equal in both settings.

## Conclusions

Patients in need of less acute treatment were considerably less common in the on-demand model, indicating a possible risk that such patients could remain unrecognized in this setting. Clinics running an on-demand model might therefore improve their psychiatric services by adding regular consultation hours. A “quasi-liaison” model, as described here, seems to be a suitable and cost-effective way of providing psychiatric care to hospital patients in small-to-medium sized hospitals that do not have associated psychiatric clinics.

## Abbreviations

CLP: Consultation/Liaison Psychiatry
DSM IV: Diagnostic and Statistical Manual of Mental Disorders IV
ICD-10: International Classification of Disease 10
LM: liaison-model
ODM: on-demand-model
